# Supplementary figures and images for: High-throughput assessment of the behavioral responses to toxic organic solvents in Caenorhabditis elegans
Source: PLoS One. 2025 Apr 17;20(4):e0311460. doi: 10.1371/journal.pone.0311460 (PMC12005522; doi:10.1371/journal.pone.0311460)

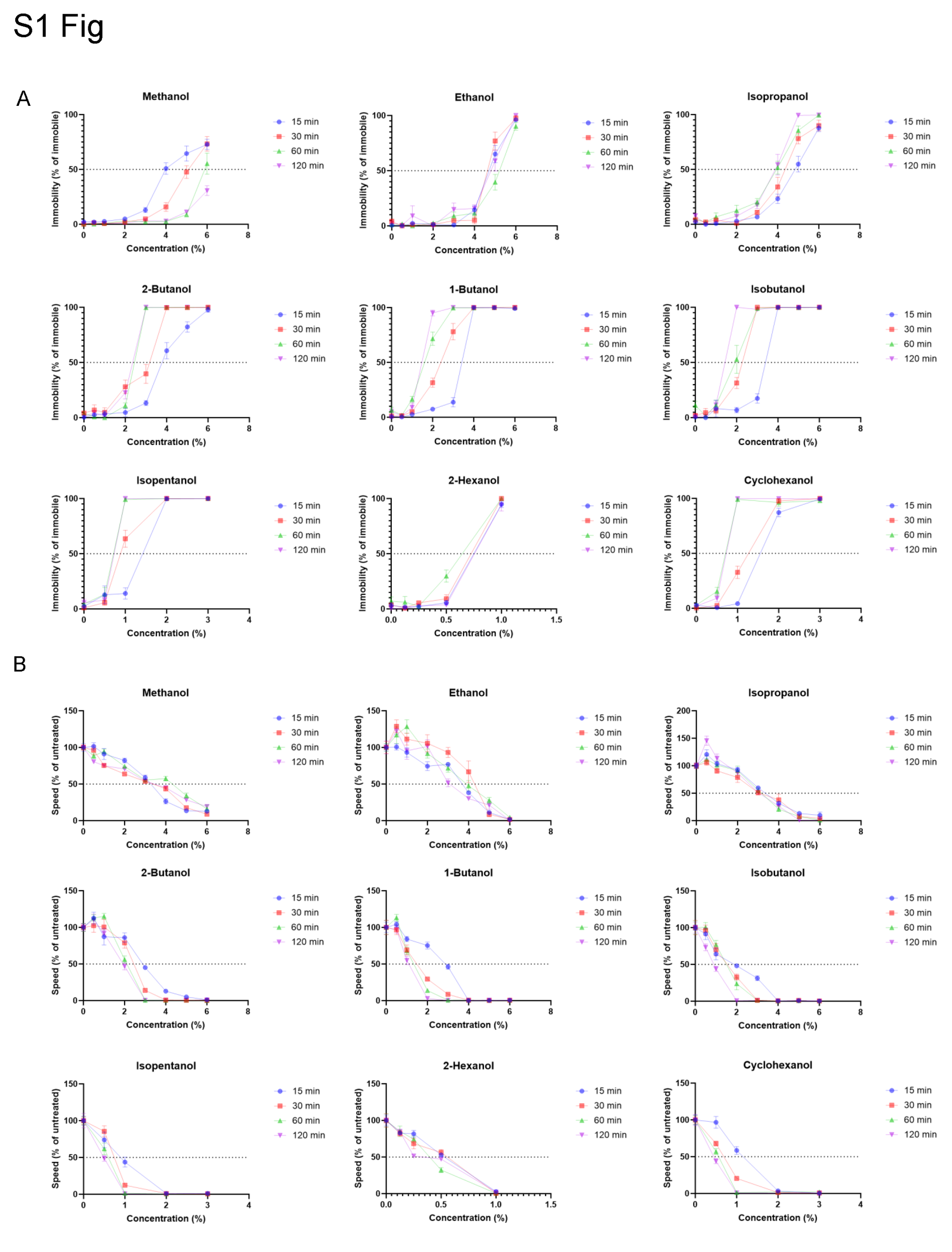

Supplement: S1 Fig — Immobility (A) and locomotion speed (B) after exposure to alcohols for 15, 30, 60, or 120 min. Each data point represents the mean ± the standard error of the mean (SEM). Locomotion speed was normalized to the average value for untreated control. (TIF) [file pone.0311460.s001.tif]

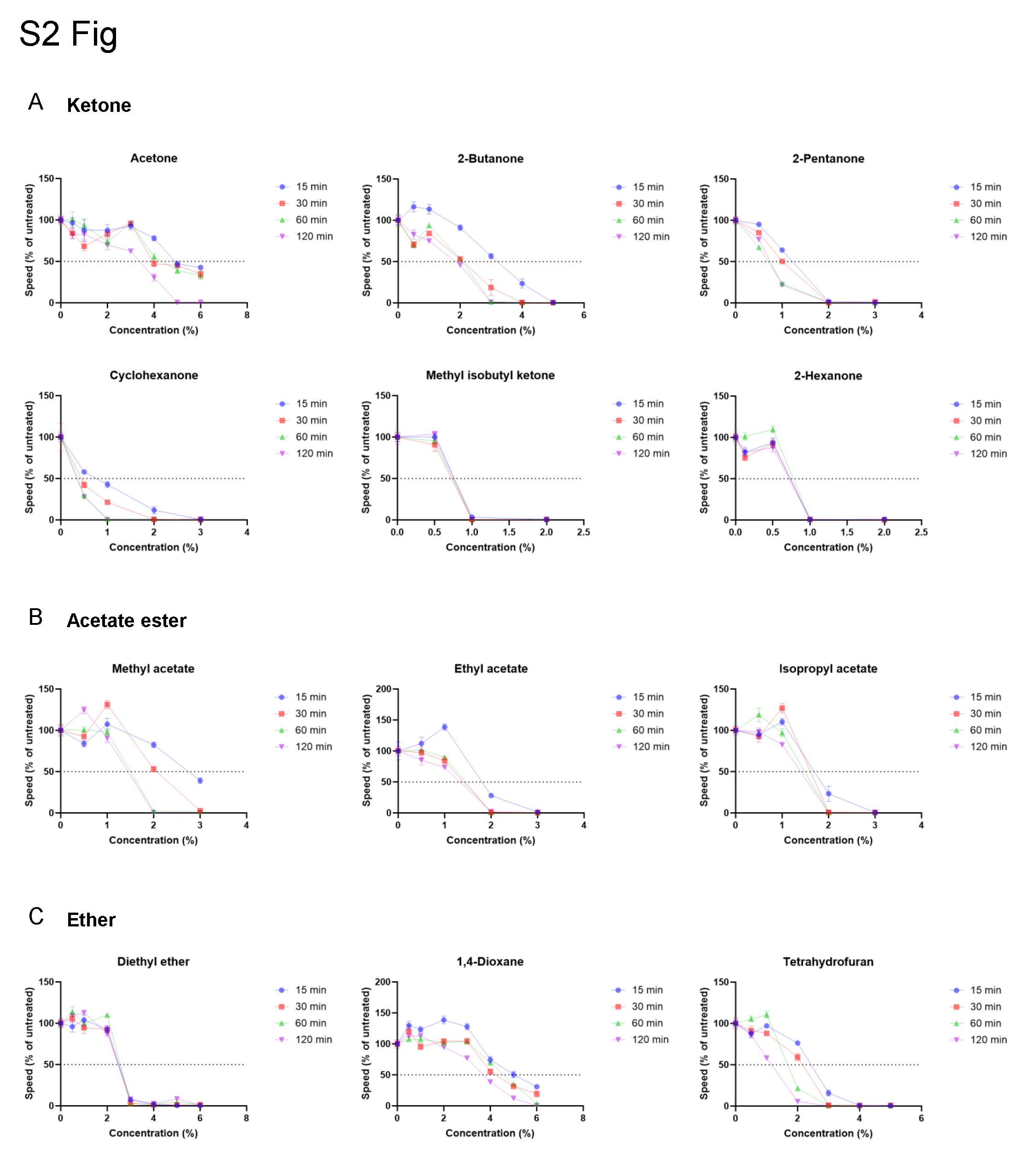

Supplement: S2 Fig — Locomotion speed after exposure to ketones (A), acetate esters (B), or ethers (C) for 15, 30, 60, or 120 min. Each data point represents the mean ± the standard error of the mean (SEM). Locomotion speed was normalized to the average value for untreated control. (TIF) [file pone.0311460.s002.tif]

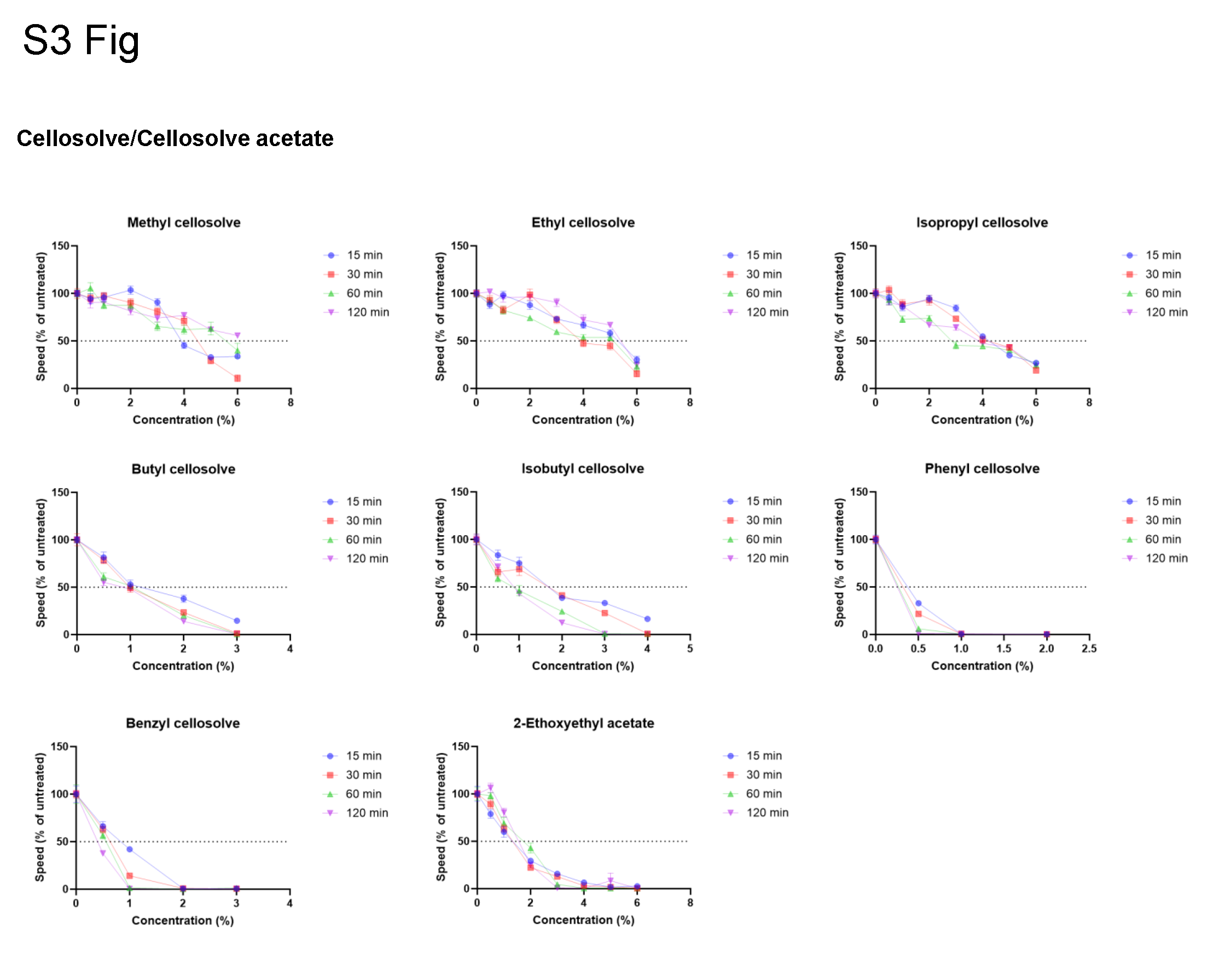

Supplement: S3 Fig — Locomotion speed after exposure to cellosolves or a cellosolve acetate for 15, 30, 60, or 120 min. Each data point represents the mean ± the standard error of the mean (SEM). Locomotion speed was normalized to the average value for untreated control. (TIF) [file pone.0311460.s003.tif]

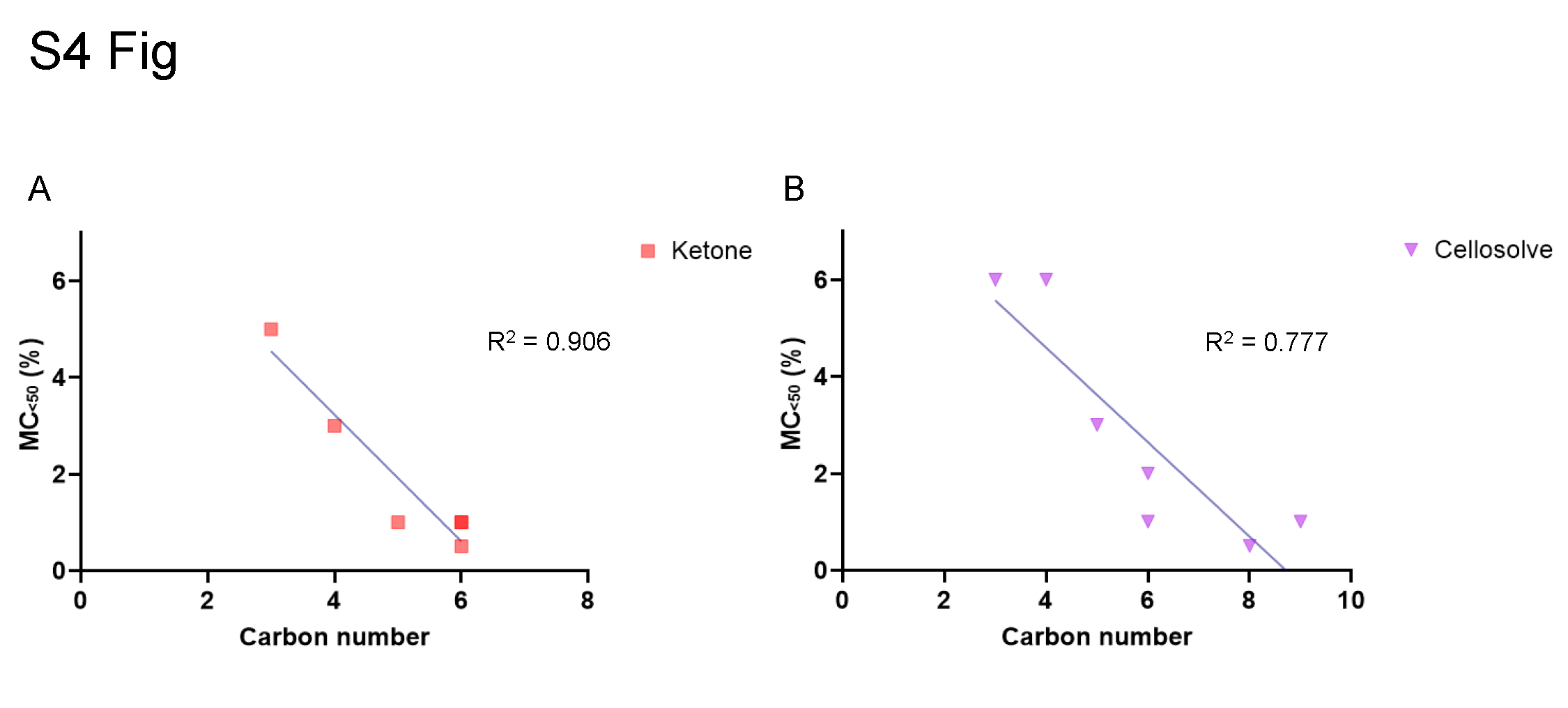

Supplement: S4 Fig — Relationship between the behavioral toxicity (MC<50) after 1 h of exposure to ketones (A) or cellosolves (B) and the carbon number of the chemicals. Locomotion speed is used as an endpoint. R2 values were determined based on simple linear regression analysis. (TIF) [file pone.0311460.s004.tif]

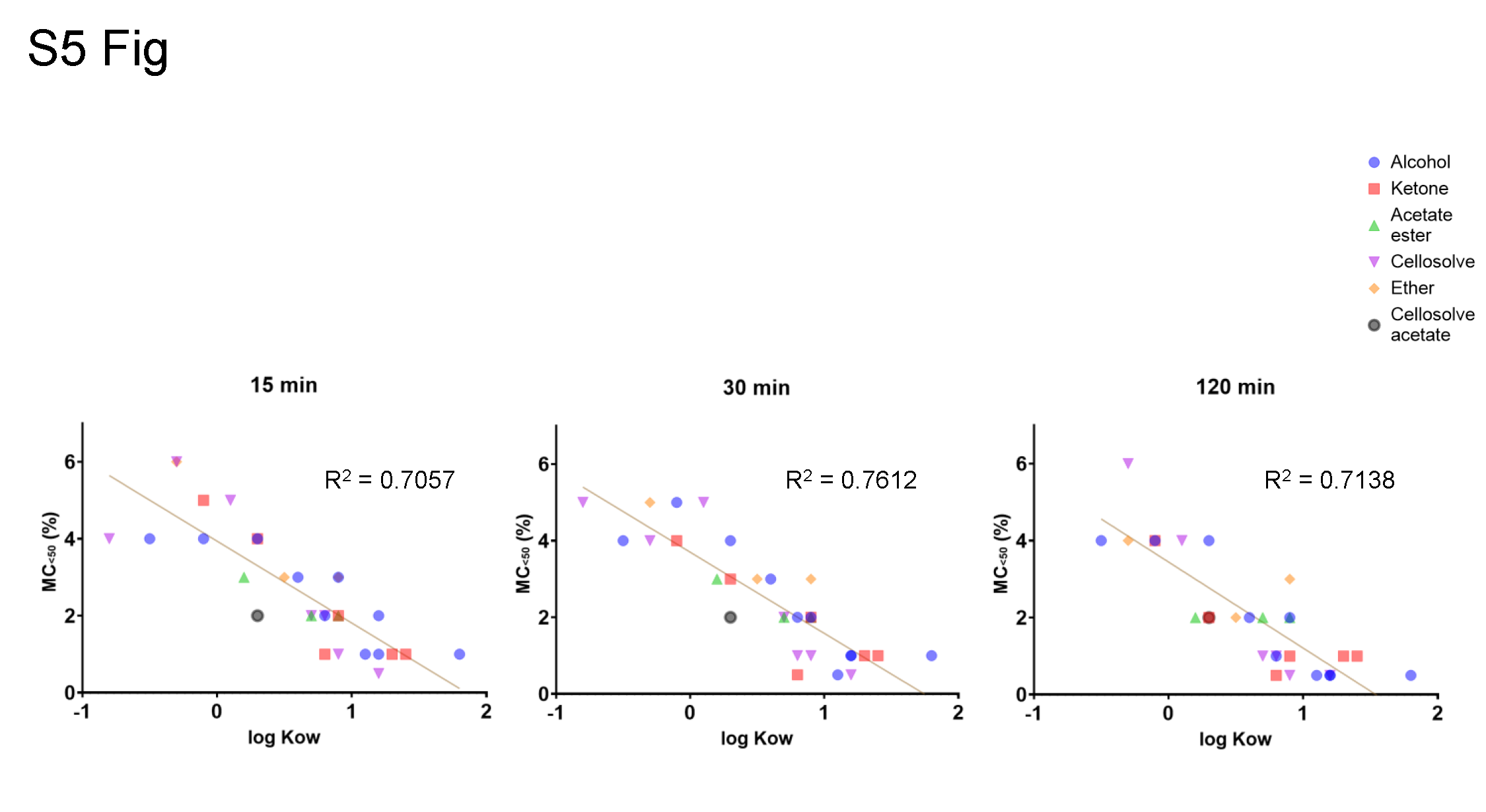

Supplement: S5 Fig — Relationship between the MC<50 after exposure to organic solvents for 15, 30, or 120 min and the octanol–water partition coefficient (log Kow), which reflects the lipid solubility of organic solvents. Locomotion speed is used as an endpoint. R2 values were determined based on simple linear regression analysis. (TIF) [file pone.0311460.s005.tif]

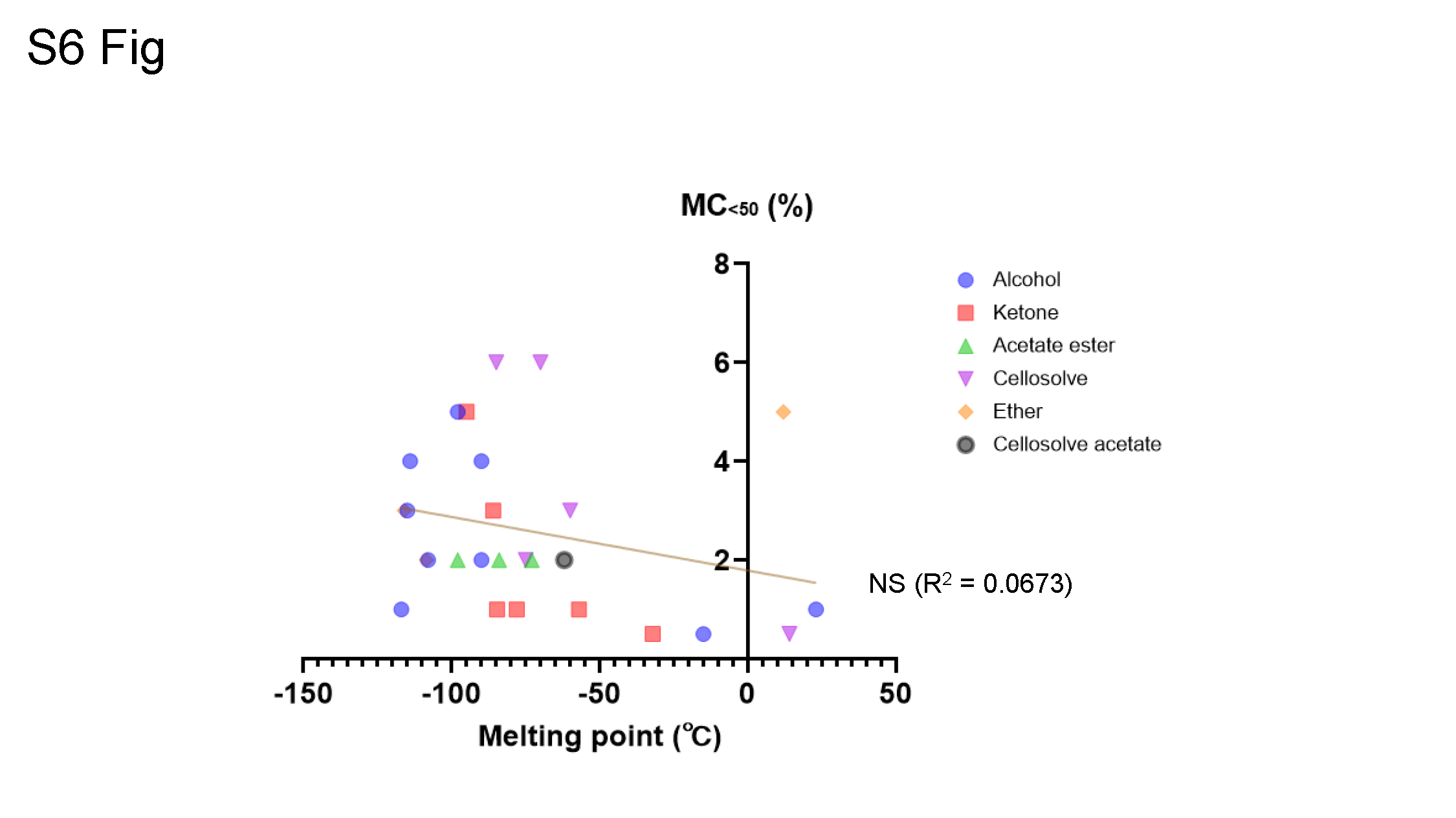

Supplement: S6 Fig — Relationship between the MC<50 after exposure to organic solvents for 1 h and the organic solvent’s melting point. Locomotion speed is used as an endpoint. P and R2 values are determined based on simple linear regression analysis, and no significant correlation is observed (P = 0.1913). (TIF) [file pone.0311460.s006.tif]

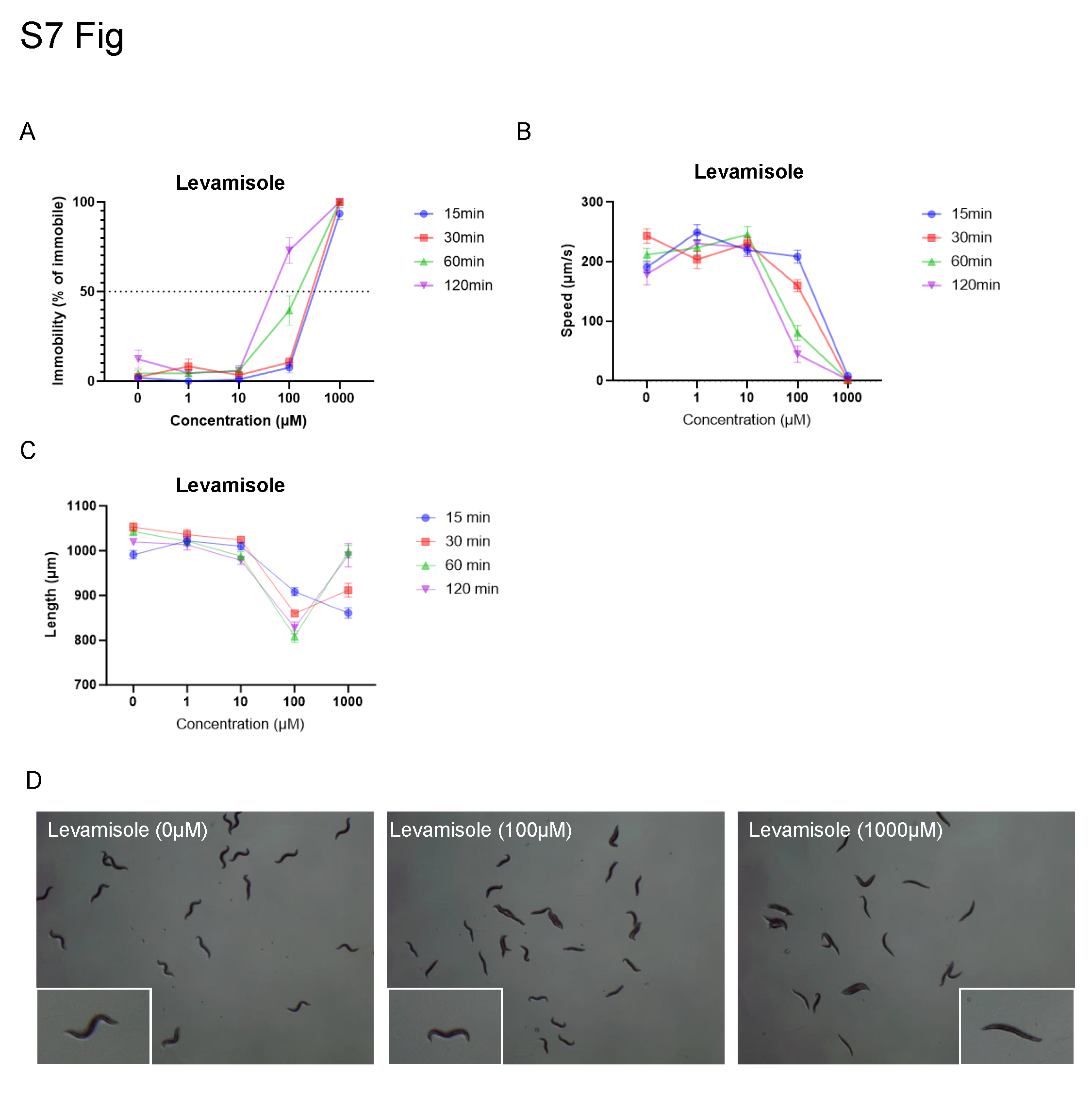

Supplement: S7 Fig — (A–C) Immobility (A), locomotion speed (B), and body length (C) after exposure to levamisole for 15, 30, 60, or 120 min. Each data point represents the mean ± the standard error of the mean (SEM). (D) Representative images of nematodes on agar plates after exposure to different concentrations of levamisole for 120 min. (TIF) [file pone.0311460.s007.tif]

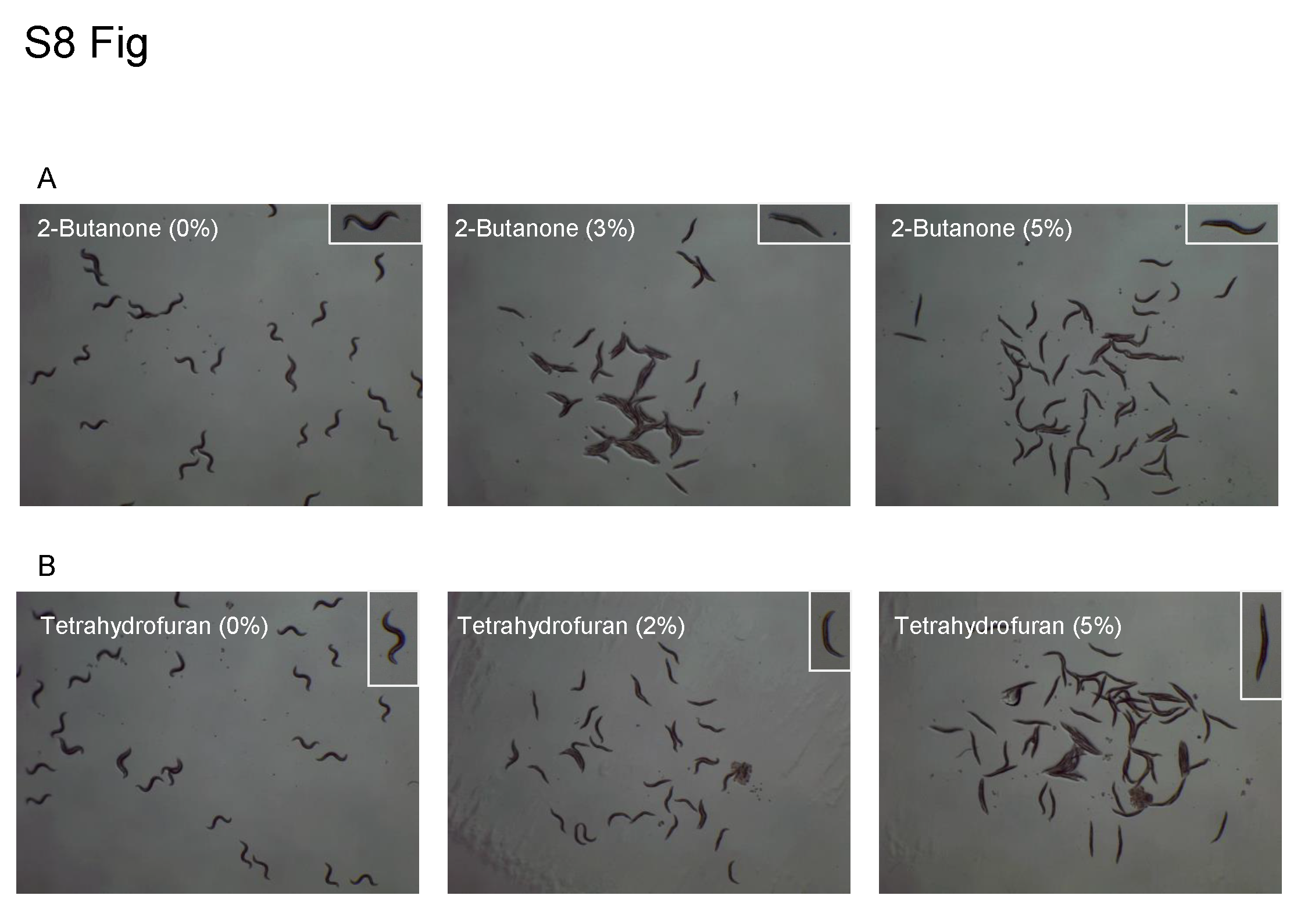

Supplement: S8 Fig — Representative images of nematodes on agar plates after exposure to different concentrations of 2-butanone (A) or tetrahydrofuran (B) for 120 min. (TIF) [file pone.0311460.s008.tif]

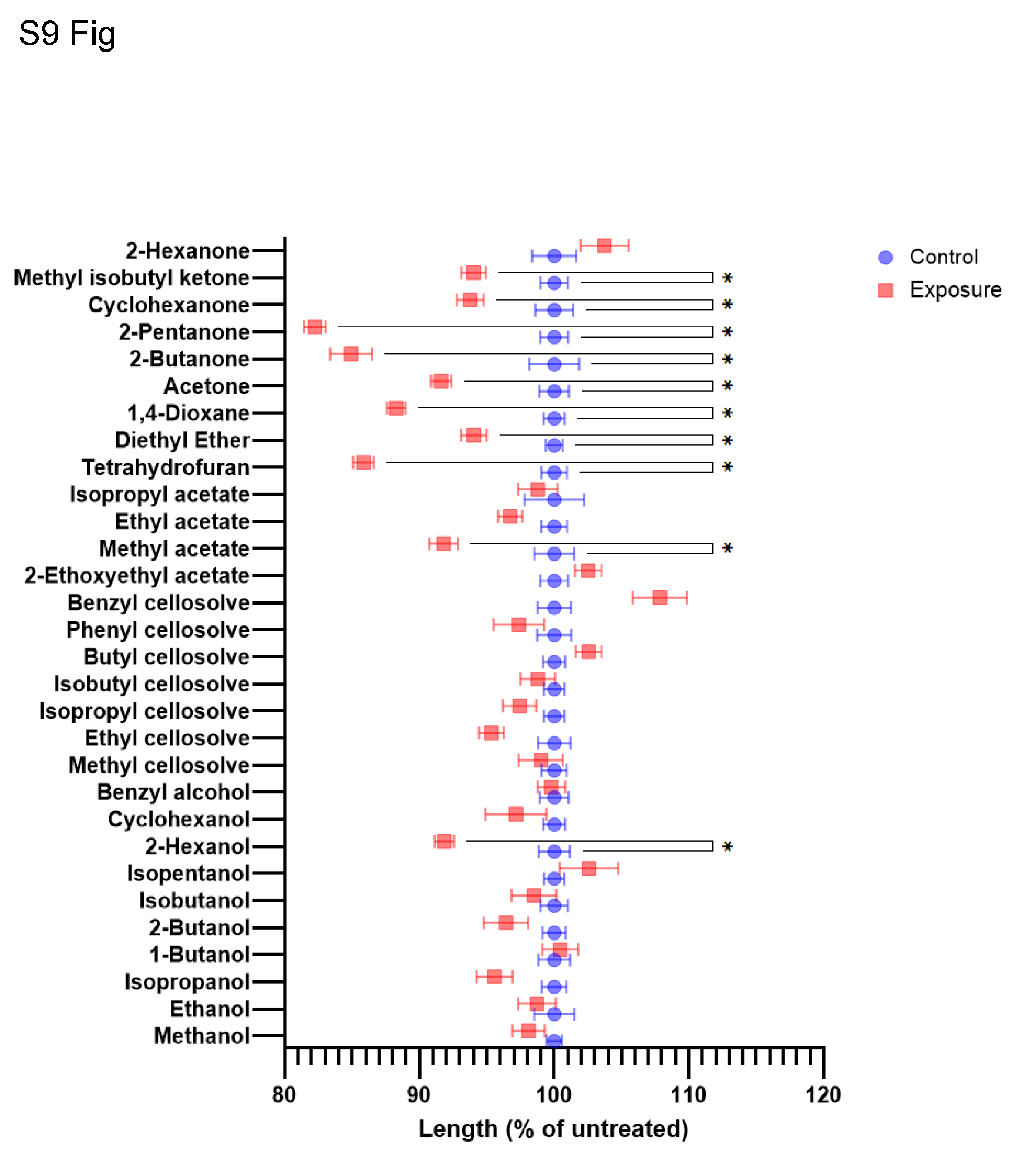

Supplement: S9 Fig — The mean values ± the standard error of the mean (SEM) of the body lengths of nematodes after 1 h exposure to organic solvents (red) or without exposure (blue). The exposure concentrations are the MC<50 values, which were determined by the locomotion speed after 1 h of exposure. Data were normalized to the average values of the no-exposure control. *P < 0.05, unpaired t-test with Holm–Sidak correction. (TIF) [file pone.0311460.s009.tif]

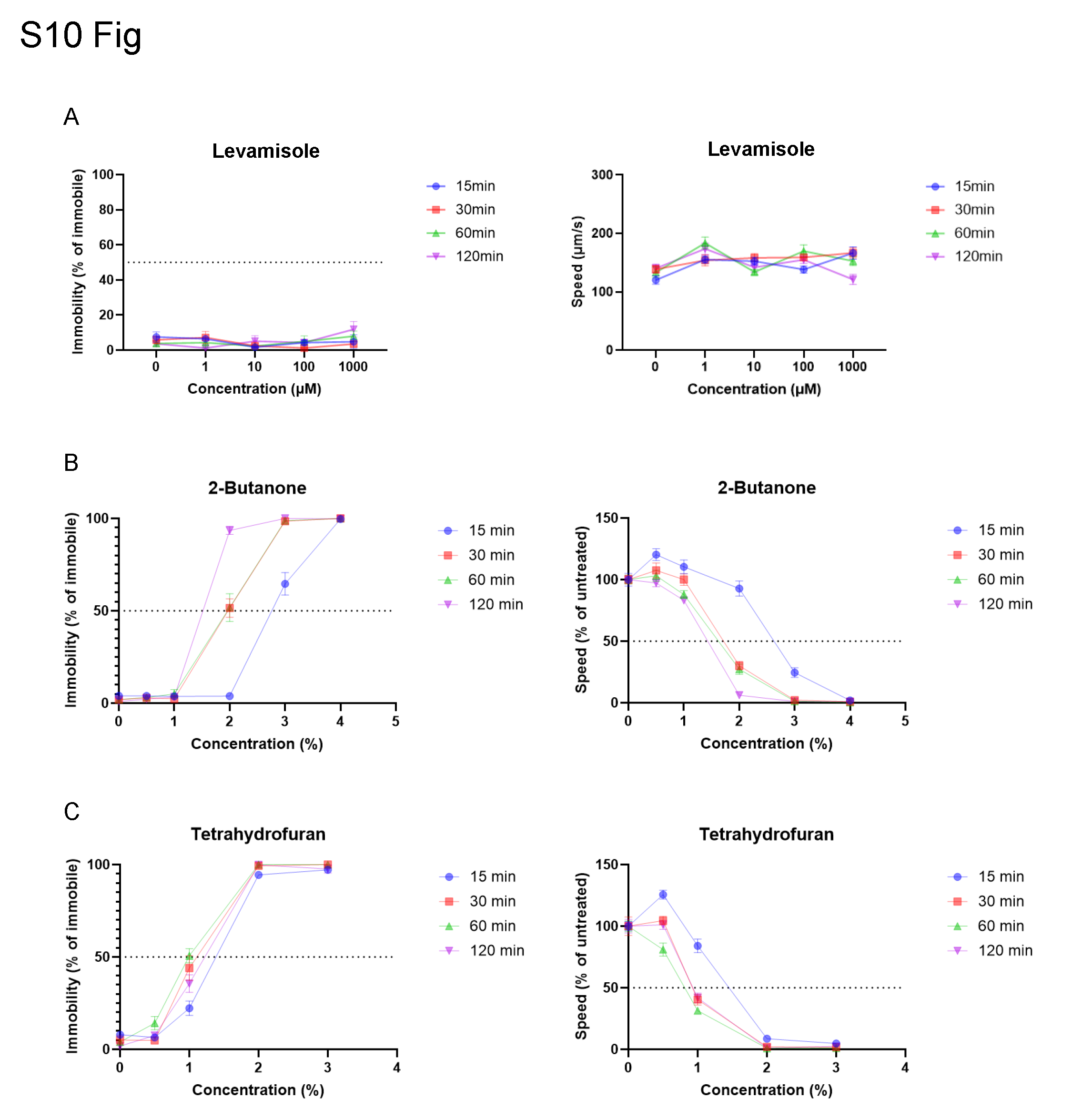

Supplement: S10 Fig — Immobility (left column) and locomotion speed (right column) of unc-29(e193) after exposure to levamisole (A), 2-butanone (B), or tetrahydrofuran (C) for 15, 30, 60, or 120 min. Each data point represents the mean ± the standard error of the mean (SEM). Locomotion speed was normalized to the average value for untreated control (B, C). (TIF) [file pone.0311460.s010.tif]

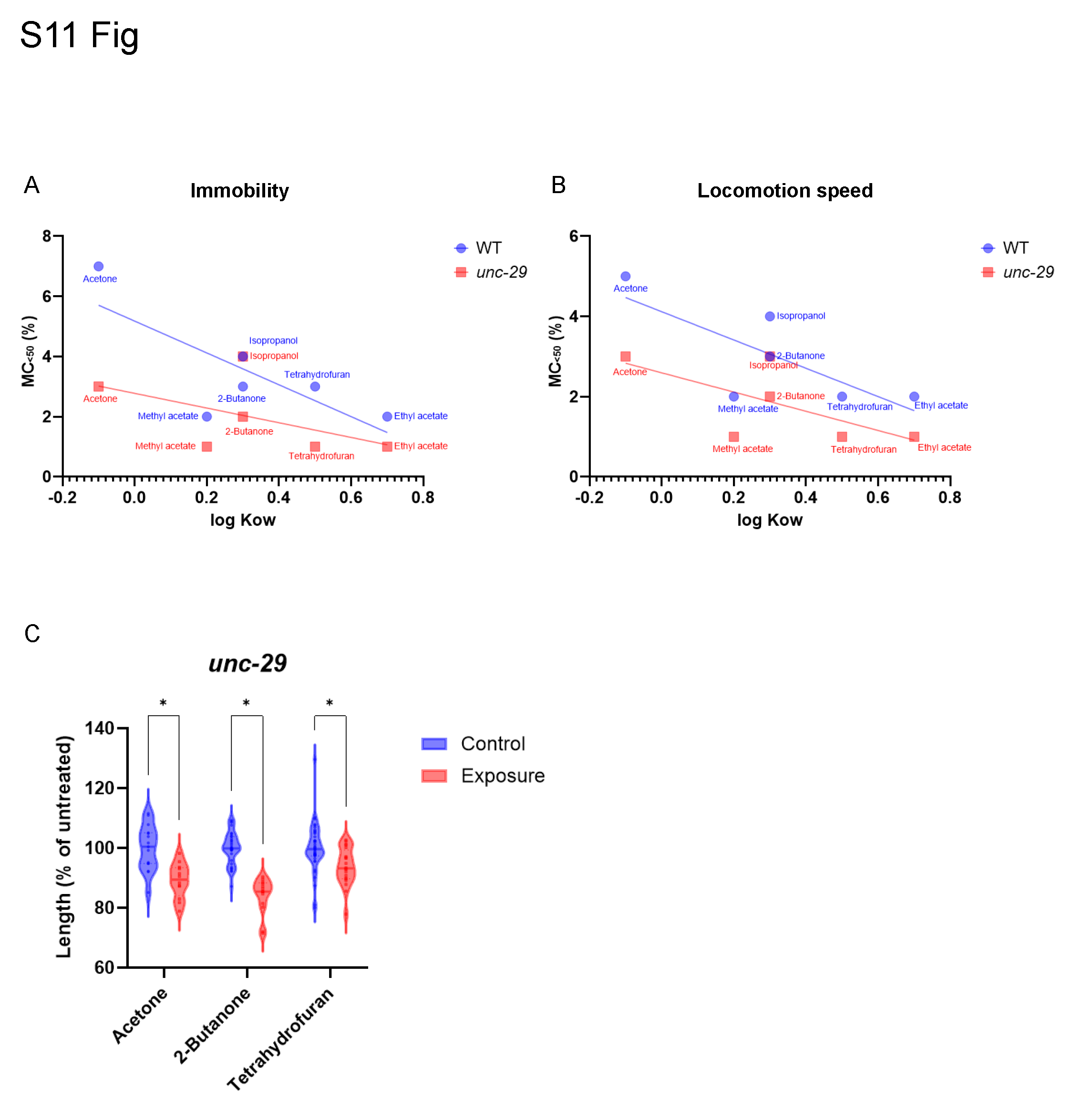

Supplement: S11 Fig — (A, B) Relationship between the MC<50 after organic solvent exposure for 1 h and the octanol–water partition coefficient (log Kow), which reflects the lipid solubility of an organic solvent. Immobility (A) and locomotion speed (B) are used as the endpoints in the wild type (blue) and unc-29(e193) (red). (C) Violin plots of the body lengths of unc-29(e193) mutants after exposure to organic solvents. The exposure concentrations are same as those used for the analysis in the wild type (Fig 3B). Data were normalized to the average values of the no-exposure control. Each dot represents the body length of a nematode after exposure (red) or without exposure (blue). *P < 0.05, unpaired t-test with Holm–Sidak correction. (TIF) [file pone.0311460.s011.tif]

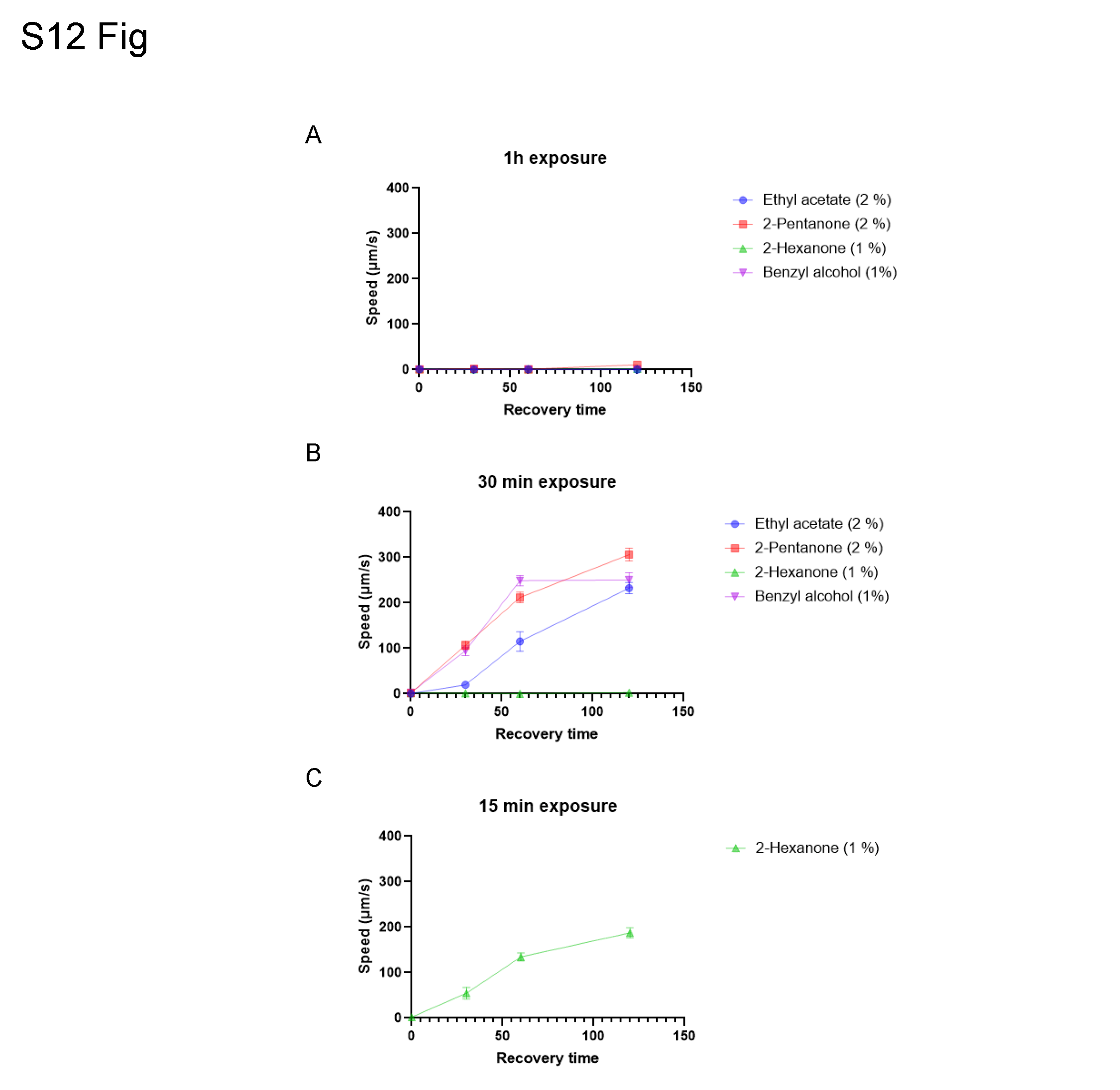

Supplement: S12 Fig — Locomotion speed after recovery in a buffer solution after exposure to an organic solvent for 1 h (A), 30 min (B), or 15 min (C) are shown. The exposure concentrations of the organic solvents are the minimum concentrations that cause complete paralysis. Each data point represents the mean ± the standard error of the mean (SEM). (TIF) [file pone.0311460.s012.tif]

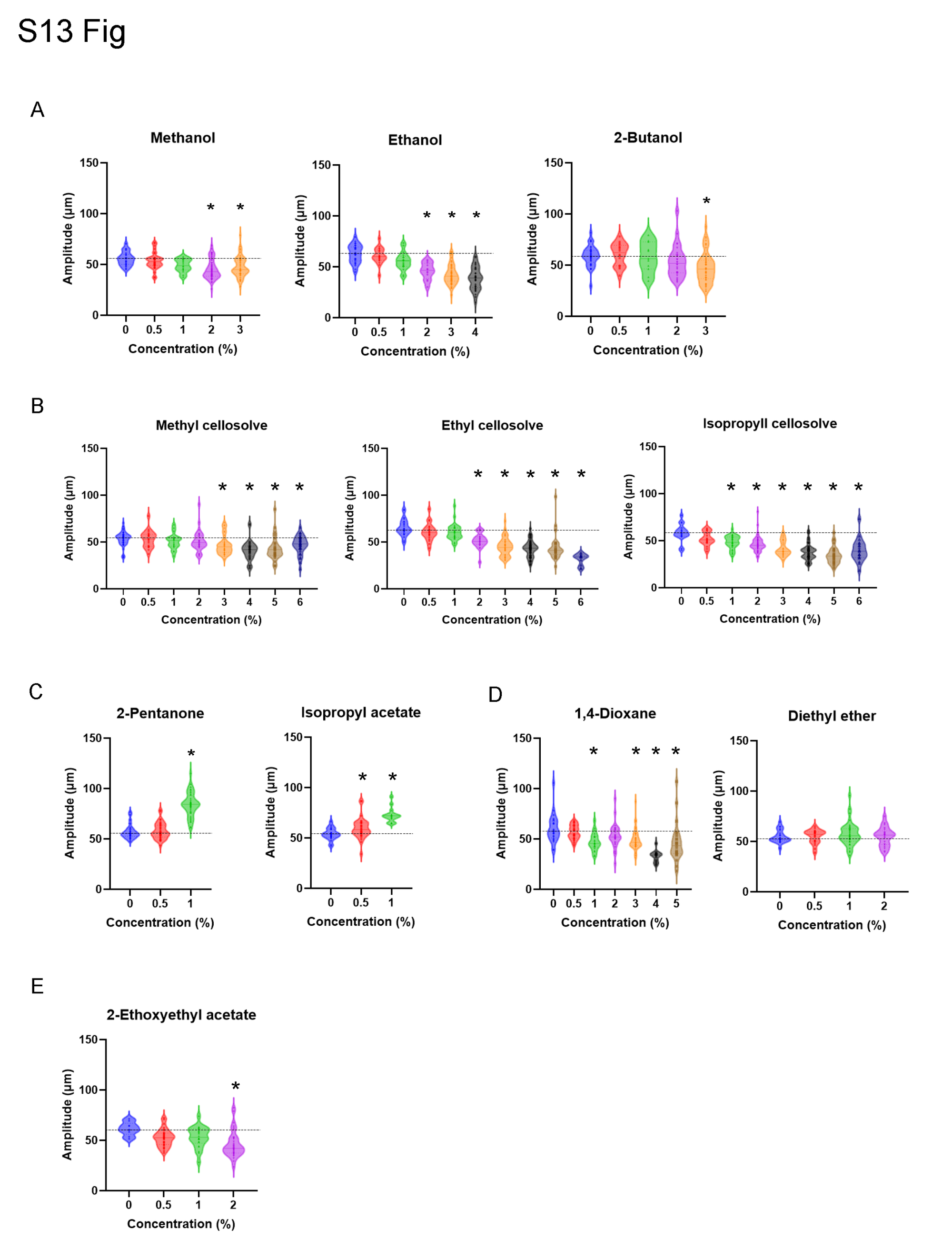

Supplement: S13 Fig — Violin plots of the amplitudes after soaking in a buffer containing organic solvents for 15 min. Each dot represents the mean amplitude during each track. *P < 0.05, one-way ANOVA with Dunnett test, compared with the no-exposure control. (TIF) [file pone.0311460.s013.tif]

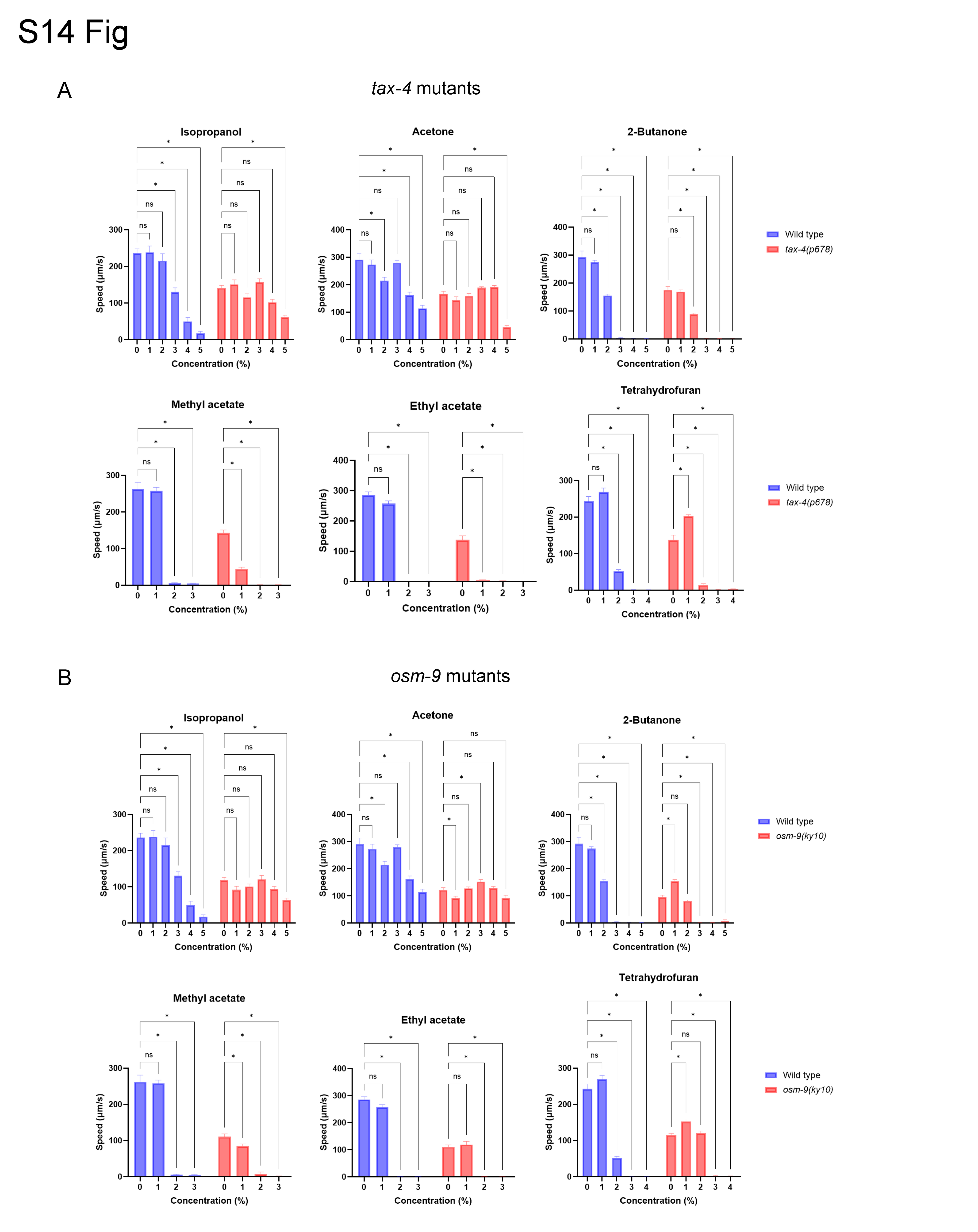

Supplement: S14 Fig — Locomotion speed of tax-4(p678) (A, red bars), osm-9(ky10) (B, red bars), and the wild-type (blue bars) nematodes after exposure to organic solvents for 1 h. Each bar represents the mean ± the standard error of the mean. *P < 0.05, one-way ANOVA with Dunnett test, compared with the no-exposure control. (TIF) [file pone.0311460.s014.tif]

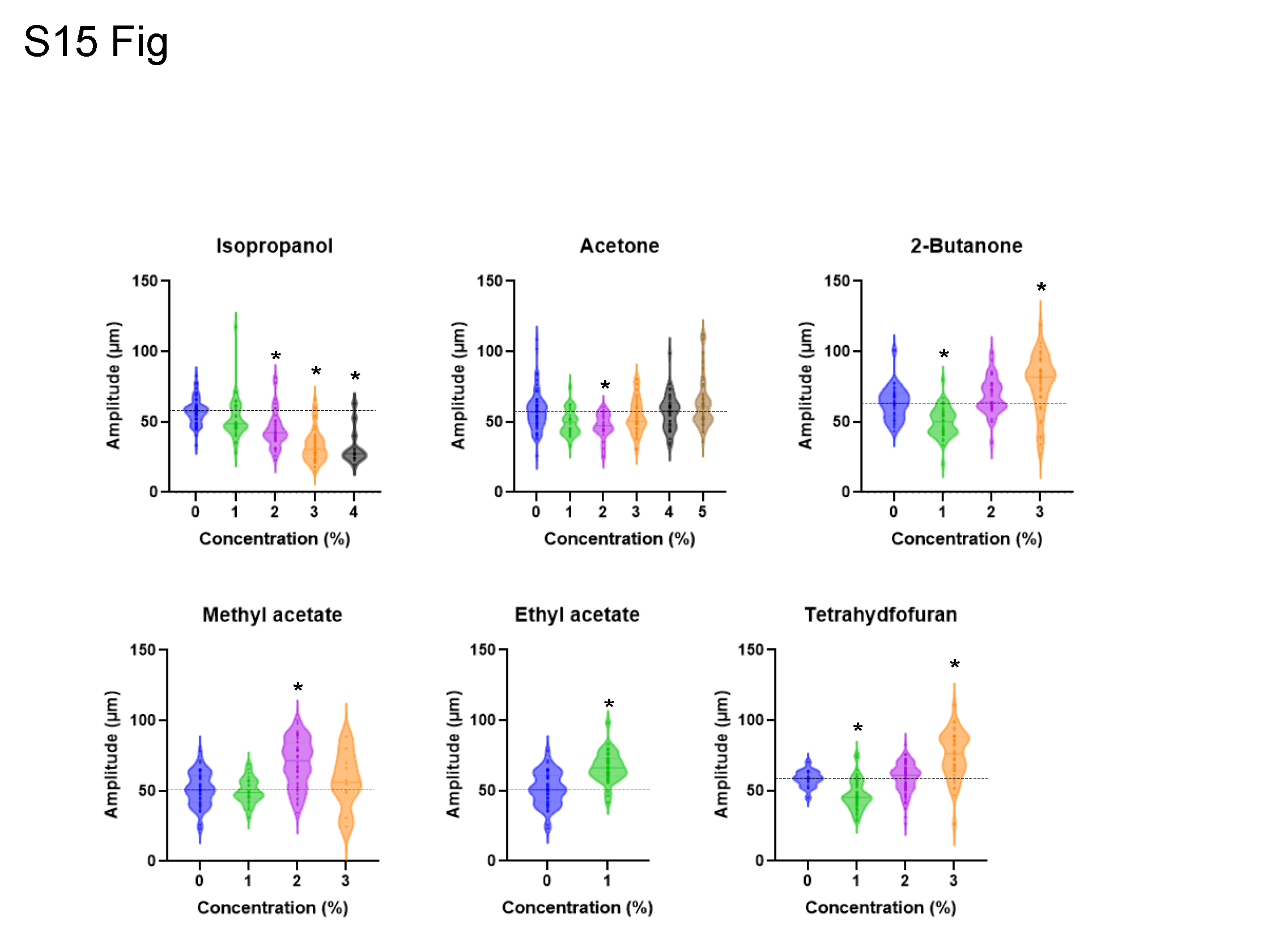

Supplement: S15 Fig — Violin plots of the amplitudes after soaking in a buffer containing organic solvents for 15 min in osm-9(ky10) nematodes. Each dot represents the mean amplitude during each track. *P < 0.05, one-way ANOVA with Dunnett test or Welch’s t-test (for Ethyl acetate), compared with the no-exposure control. (TIF) [file pone.0311460.s015.tif]

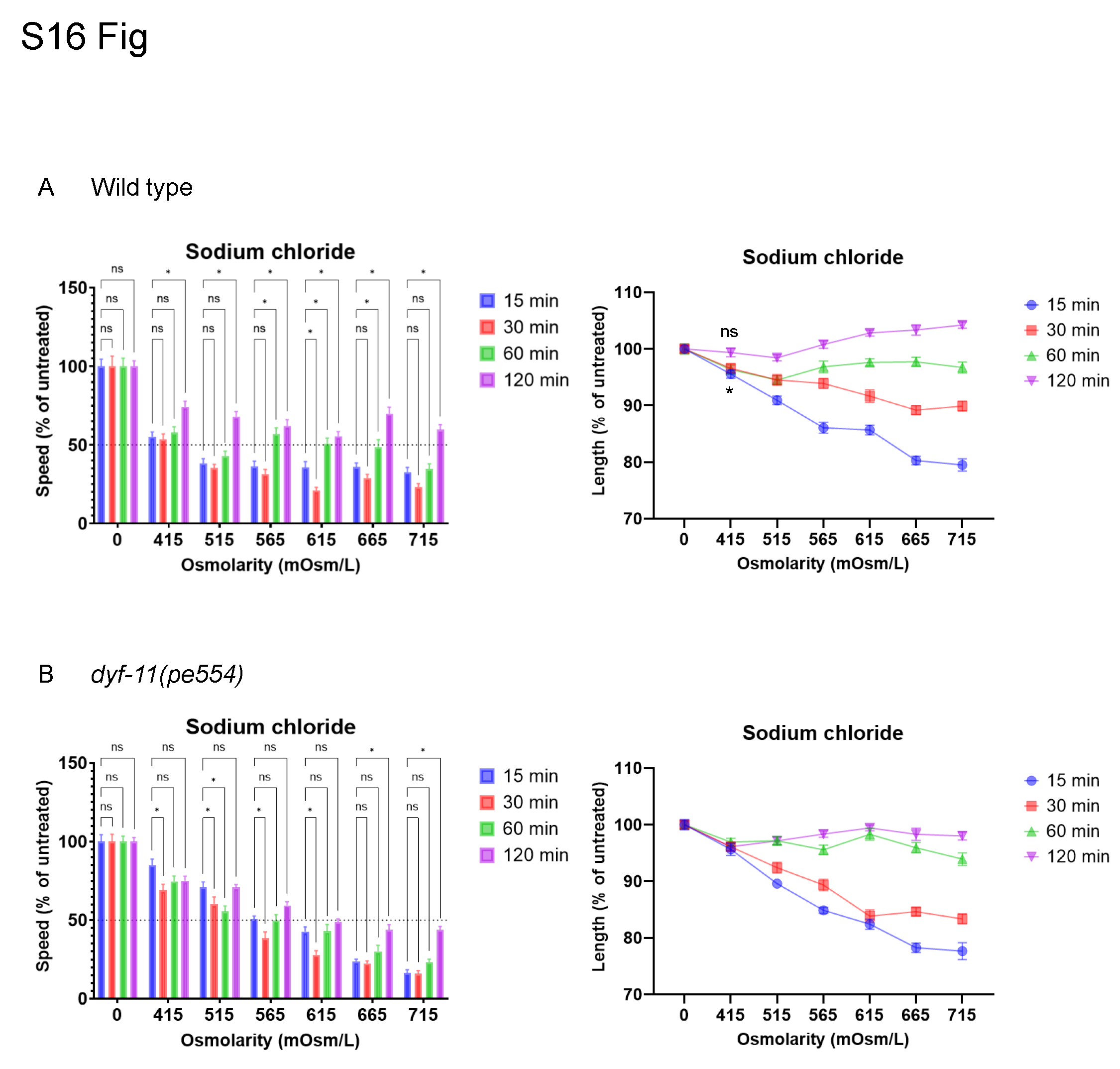

Supplement: S16 Fig — Locomotion speed (left) and body length (right) of the wild type (A) and the dyf-11(pe554) mutant (B) after exposure to sodium chloride for 15, 30, 60, or 120 min. Each bar or data point represents the mean ± the standard error of the mean. *P < 0.05, one-way ANOVA with Dunnett test, compared with the no-exposure control. (TIF) [file pone.0311460.s016.tif]

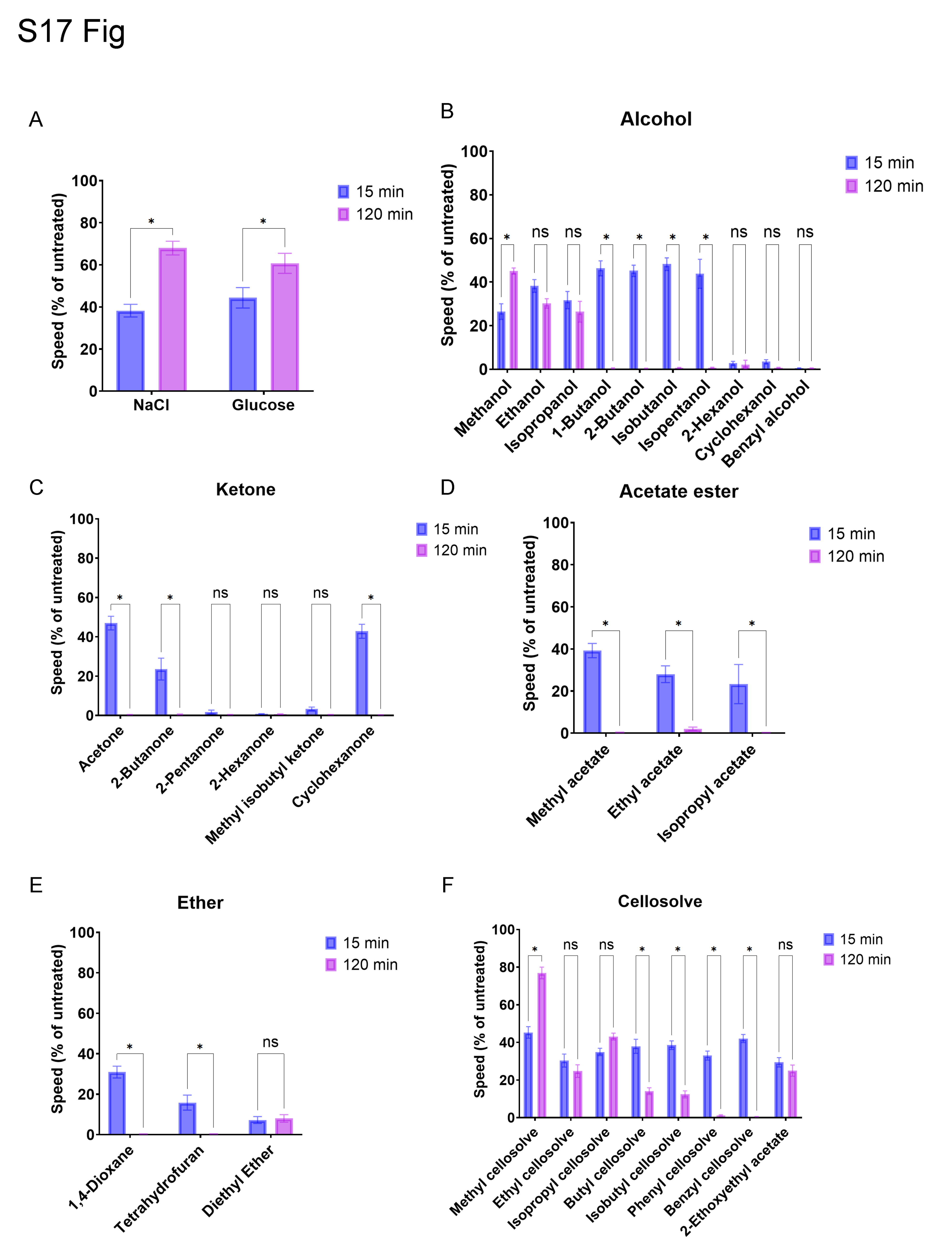

Supplement: S17 Fig — Locomotion speed after exposure to indicated chemicals for 15 or 120 min. Each bar represents the mean ± the standard error of the mean. *P < 0.05, unpaired t-test with Holm–Sidak correction. Locomotion speed was normalized to the average value for untreated nematodes. (TIF) [file pone.0311460.s017.tif]

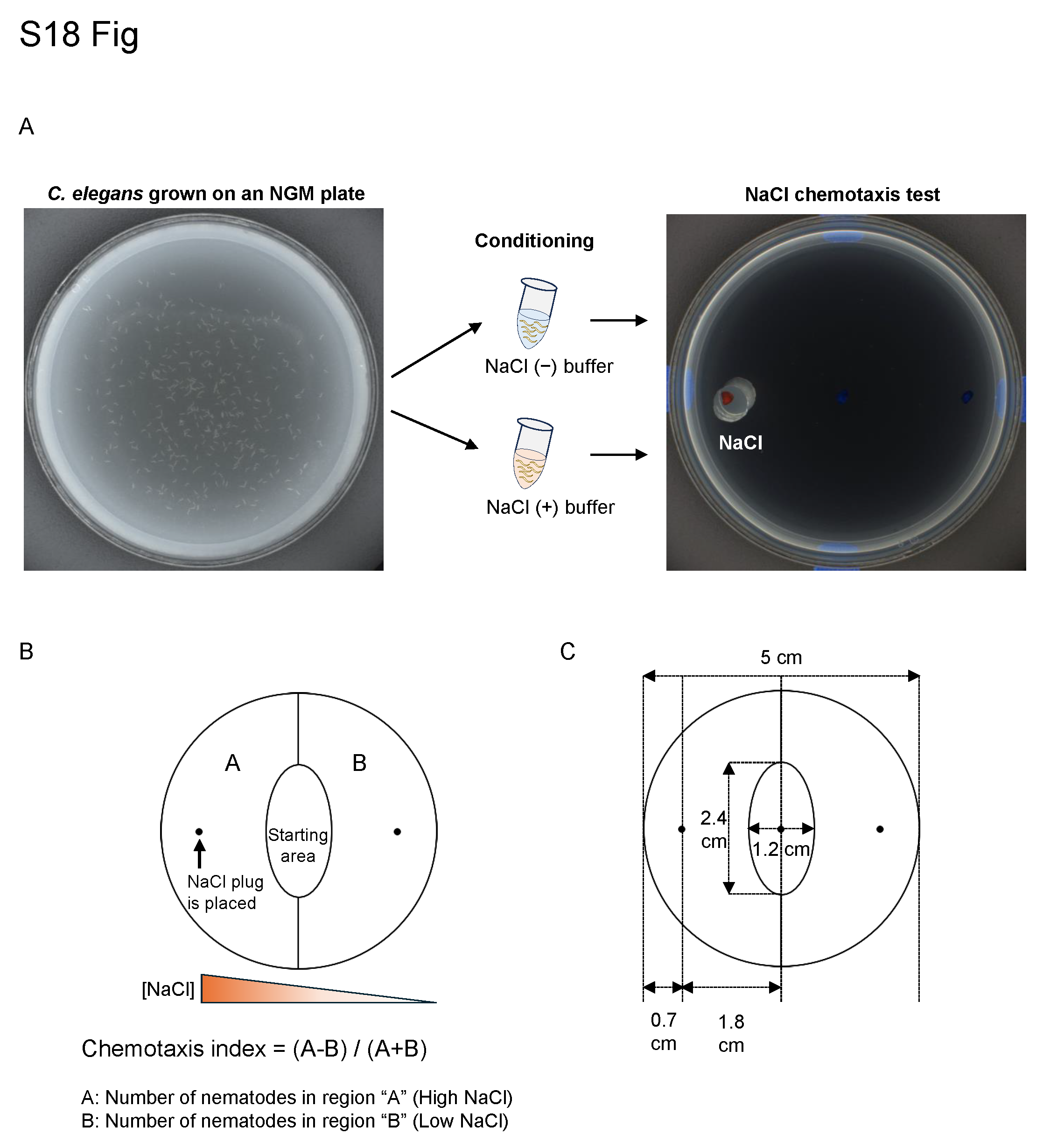

Supplement: S18 Fig — (A) Procedure for the salt chemotaxis learning assay. (B) Format for the salt chemotaxis test and calculation of the chemotaxis index. (C) Size of a chemotaxis test plate. (TIF) [file pone.0311460.s018.tif]

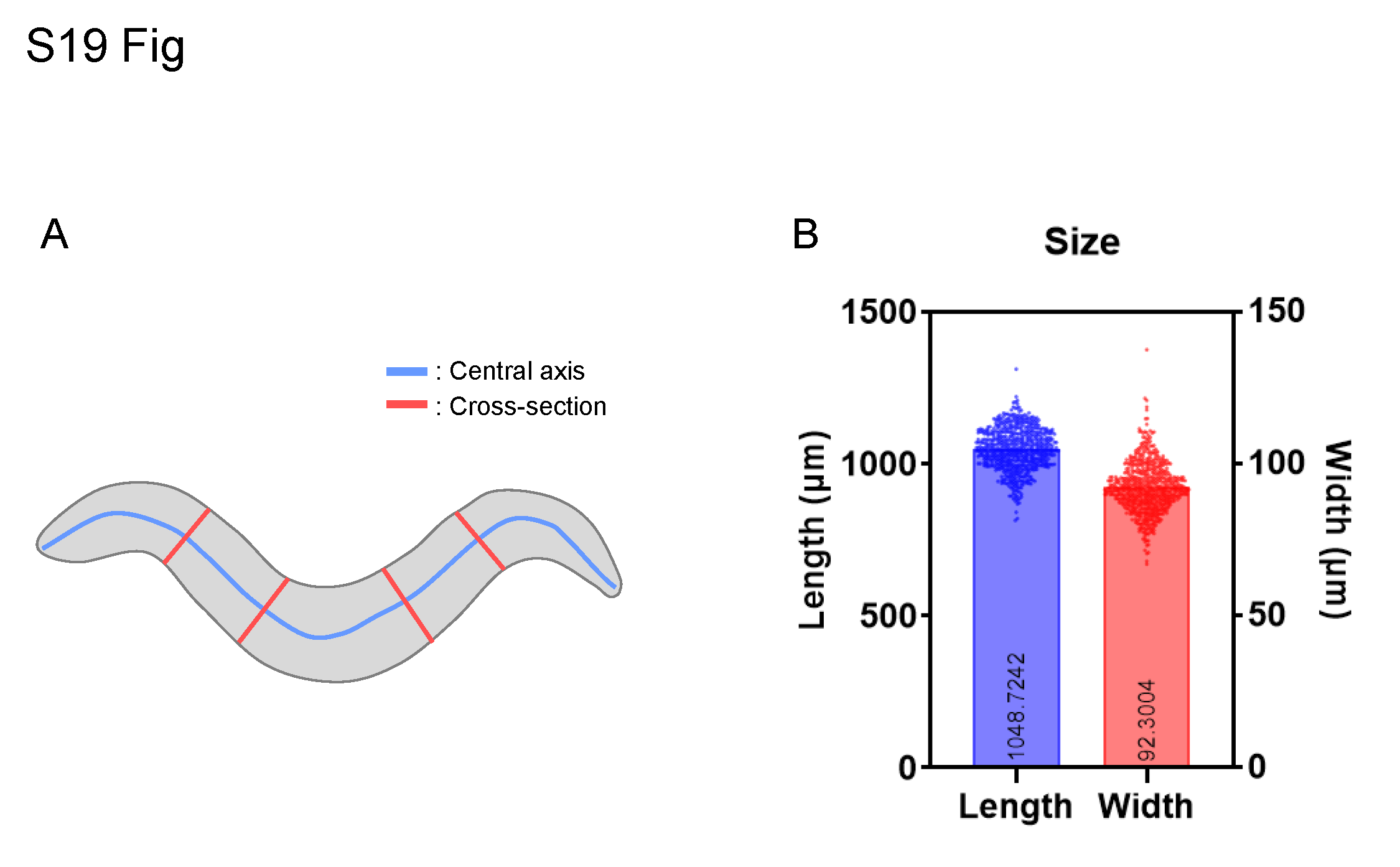

Supplement: S19 Fig — (A) The body length of a nematode was determined as the length from head to tail along the central axis (blue line). The body width was determined as the average length of cross-sections (red lines) over the entire body. (B) The average body length (left, 1049 µm) and the average body width (right, 92.30 µm) were determined based on body lengths and widths of 650 nematodes, calculated using Wormlab. (TIF) [file pone.0311460.s019.tif]
