# Supplementary material for: High-throughput assessment of the behavioral responses to toxic organic solvents in Caenorhabditis elegans
Source: PLoS One. 2025 Apr 17;20(4):e0311460. doi: 10.1371/journal.pone.0311460 (PMC12005522; doi:10.1371/journal.pone.0311460)
Supplement: Attachment — Submitted filename: Response to Reviewers.docx [file pone.0311460.s030.docx]

**Response to Reviewers**

Thank you for reviewing my manuscript and providing me with constructive and insightful comments on the manuscript. I performed experiments to address the reviewers’ concerns and thoroughly revised the manuscript according to the reviewers’ comments. Furthermore, the manuscript has been edited for English language, grammar, punctuation, and spelling by a proofreading service, Enago. Below is my point-by-point response to the comments.

**Response to Reviewer #1**

**Major issues:**

*1. The author repeatedly uses the phrase “adverse effects” and clarify their assumption that responses to the solvents represent a passive response of the solvents on the neurons and/or muscles. However, they do not consider to what extent these responses may reflect a mixture of that as well as the following: a) active attempts by the worm to escape the toxin by increasing speed via body curvature, b) active attempts to slow down due to attraction to the solvent (see PMID: 8348618), c) passive yet reservable responses by the worm to dehydration by the solvent (see PMID: 15166144). Rather than “adverse”, I’d recommend a neutral term such as behavioral response or change. Even for salt attraction plasticity, it seems adaptive rather than maladaptive to not form an association when danger is apparent. So, this isn’t obviously an adverse effect in my opinion.*

I explored what extent the locomotion changes after organic solvents depend on sensory responses and dehydration due to hyperosmolarity.

Regarding the sensory responses, I examined behavior using mutants of *tax-4* and *osm-9,* which function redundantly in chemosensory responses. Please see the results (line 448-486), figures (Figure 2F, 5D, 6 and Supporting Figure 14-15), and discussion (line 669-685) in the revised manuscripts. Based on these experiments, I obtained suggestions as follows.

A) Chemosensory input of organic solvents may affect locomotion speed after prolonged exposure to organic solvents, where extent and direction of the effects are variable dependent on the organic solvents.
B) TAX-4-mediated signaling is required for increased amplitude of the body bends during locomotion after exposure to organic solvents, such as ketones and acetate ester. This result suggests that the sensory response through TAX-4-mediated signaling may promote an increase in the amplitude after organic solvent exposure.
C) I examined exposing time of the solvents required for an increase in the amplitude and found that more than 5 min exposure of 2-butanone is required for a prolonged increase in the amplitude of the body bend. The finding is consistent with the notion that prolonged sensory input via TAX-4-mediated signaling induces long lasting increase in the amplitude of body bending. Further, the result suggests that the phenomenon reported in this study is different from the quick behavioral response following quick activation of chemosensory neurons, which are reported in the phenomena, such as chemotaxis to odorant (PMID: 18817734).

Regarding dehydration, I examined the effects of exposure to NaCl and glucose, which are known as less toxic water-soluble chemicals, on locomotion. Please see the results (line 496-549), figures (Figure 7, Supporting Figure 16-17), and discussion (696-716) in the revised manuscript. Based on these experiments, I obtained results and suggestions as follows.

A) Fifteen minutes exposure to >515 mOsm solution containing NaCl or glucose caused a marked decrease in locomotion. The decrease in locomotion speed after NaCl or glucose exposure was recovered after a subsequent 105 min exposure (for a total of 120 min exposure) in the same solution.
B) The MC_<50_ (mM) values, the minimal molar concentration required for >50% reduction in locomotion speed, of organic solvents with high lipid solubility were much lower than that of glucose, indicating that these organic solvents were more effective on decrease in locomotion speed than glucose. For example, the MC_<50_ (mM) value of benzyl alcohol is more than 10 times smaller than that of glucose. The result suggests that organic solvents with high lipid solubility reduce motility differently from dehydration due to hyperosmolarity.
C) On the contrary, the MC_<50_ (mM) values of organic solvents with lower lipid solubility, such as methanol, ethanol and acetone, were higher than that of glucose. However, unlike NaCl and glucose, decreased motility caused by 15 min exposure of each of 28 organic solvents was not recovered by a subsequent 105 min exposure in the same solution, implying that the organic solvents affect motility in a manner different from that caused by dehydration due to hyperosmolarity. The two exceptions among 30 organic solvents were methanol and methyl cellosolve, where the reduced motility after 15 min exposure was significantly recovered by a subsequent 105 min exposure in the same solution. I concluded that it is possible that reduced motility occurs after exposure of organic solvents with high hydrophilicity, such as methanol and methyl cellosolve, at least in part by dehydration due to hyperosmolarity.

I also explored what extent salt chemotaxis learning defects depend on perception of organic solvents by odor-sensing chemosensory neurons. Please see the results (line 592-605) and figures (Figure 8C) in the revised manuscript. I found that organic solvents cause defects in salt chemotaxis learning even in the *odr-3* mutant, which is highly defective in most chemosensory responses to volatile chemicals. Although I cannot rule out the involvement of chemosensory signaling independent of ODR-3, the finding suggests that organic solvents may affect behavioral plasticity through mechanisms other than chemosensory signaling. Meanwhile, it should be further explored for the molecular and cellular mechanism to understand the effect of organic solvents on chemotaxis learning in the future.

Based on these findings, I reconsider how to explain the effects of organic solvents on several behaviors.

A) I used “adverse effect” for decrease in locomotion speed after organic solvent exposure, because organic solvents, especially those with high lipid solubility, did not depend on chemosensory signaling and dehydration.
B) On the other hand, it was suggested that the effects on amplitude of the body bends depend on chemosensory functions. The mechanisms of action of organic solvents on salt chemotaxis learning is obscure. Therefore, I simply explained those effects using phrases, such as “behavioral response”, “effect”, “affect”, and “suppression”.

*2. How much of chemicals penetrate the worm given the amount, duration of treatment, and buffer? I don’t expect the author to make measured estimates for internal concentrations for each of the 30 solvents and at each dose. But it would be appropriate for them to measure some and to cite other papers that have measured these parameters already for some chemicals (e.g. gas chromatography or colorimetric assays) (see PMID: 22486589). For the goal of their work to be relevant to human toxicity, it is important to know if internal concentrations that elicit behavioral endpoints in worm relate to toxicity in human tissues.*

As pointed out by the reviewer, it is important to understand the internal chemical concentrations that cause toxic effects on worms to compare with the toxicity on mammals or humans. First, I read the Alaimo et al., 2012 paper (PMID: 22486589) which is introduced by the reviewer. According to the methods in this paper, I performed experiments to determine the estimated internal concentrations of ethanol after exposure to 500 mM ethanol in a buffer solution for 10 or 50 min using colorimetric assay. Please see the result (line 319-328 and Figure 2E) and the method (line 222-243 and Supporting Figure 19). The internal ethanol concentration after 50 min exposure was similar to that determined in the Alamio et al. paper, in which worms were exposed to the same concentration of ethanol for the same exposing time on agar plates (80.1 ± 12.7 mM, present study vs. 89.3 ± 8.8 mM, Alaimo et al., 2012). Meanwhile, the internal ethanol concentration after 10 min exposure was lower in the present study compared to that in the Alamio et al. paper (25.7 ± 3.03 mM, present study vs. 67.5 ± 7.1 mM, Alaimo et al., 2012). From this experiment, I confirmed that ethanol indeed penetrates the worm’s body by the procedure in this study. The result also suggests the difference in time course of penetration between the present study and the Alamio et al. paper. This difference might be due to the difference in exposure methods, exposure on an agar plate in the Alamio paper and in buffer solution in the current study.

*3. The author says that the site of action of toxicants have not been extensively studied (line 77), but it has for many chemicals. The Morgan lab has published many papers on how solvents act or do not act in expectation with the Meyer-Overton rule (please look for many including this one from 1995, “Overall, we think that drawbacks of C. elegans are out-weighed by the animal’s impeccable adherence to the Meyer-Overton relationship, the reversibility of our anesthetic endpoint, the qualitative behavior of the animals in various anesthetic agents, and the maintenance of a cut-off effect." PMID: 8749805, also 1990 PNAS paper PMID: 2326259). The Morgan lab identified a broad array of genes that are required to respond to specific solvents, breaking the M-O rule. Likewise, many papers have been published on protein and lipid targets of ethanol (and toluene) with quantitative analysis of worm posture and motion that are not cited and put into context here (e.g. many, but see PMID: 14675531, PMID: 15182714, PMID: 21945072, PMID: 22574115, PMID: 25342716). A suggestive amino acid target for ethanol was shown in PMID: 25031399. The author has conducted a wide range of experiments. Please put your hard work into context.*

I really appreciate the instruction of important literature. I read these papers and added the background about the toxicity study on organic chemicals in the introduction section (line 87-106) and the discussion section (line 757-759). Further, in the introduction section the main subject of this paper was emphasized (line 107-112) and in the discussion section the limitation of this study and the future perspective were mentioned (line 763-766) to put my work into the context.

*4. Repeating an important idea above, just because the worm shrinks and recovers its body size with levamisole exposure, doesn’t mean that if reversable body shrinkage is also observed with a solvent that it happens the same way. The author shows no experimental evidence that the underlying mechanisms are the same. However, there are ways to do this by testing mutants deficient in an array of muscle receptors or ion channels. I worry to what extent this shrinking reflects the worm becoming dehydrated. Would osr-1 or an aquaporin mutant resist this shrinkage? Also, please discuss your UNC-29 results and body shrinkage in context with PMID: 25342716.*

I appreciate the insightful comments of the reviewer. First, to avoid confusion I deleted the description “These results imply that low molecular ketones and ethers promote muscle contraction followed by muscle relaxation similar to that of levamisole.” (line 302-304 in the 1st draft). In the revised manuscript I added the results of body shrinkage in the unc-29 mutant after exposure to ketones and tetrahydrofuran (Supporting Figure 11C) and clearly stated that “These results suggest that low-molecular-weight ketones and ethers reduce locomotion speed and body size in a UNC-29 independent manner.” (line 375-376). Further, in the discussion section I mentioned that “The finding that the unc-29 mutant exhibited body shrinkage after exposure to short-chain ketones or ethers suggests that an unknown molecule(s), other than UNC-29, is a major effector of body shrinkage caused by organic solvents.” (line 656-659). As pointed out by the reviewer, ion channels or receptors expressed in the muscle might be related to the effect of ketones and ethers on the body size. I added discussion about this possibility (line 659-663).

Regarding dehydration due to hyperosmolarity, I compared body shrinkage phenotypes after exposure to less toxic water-soluble chemicals, NaCl and glucose, with organic solvents, 2-butanone and tetrahydrofuran. I found that >415 mOsm NaCl solution or >460 mOsm glucose solution caused significant body shrinkage after 15 min exposure and subsequent 105 min exposure (total of 120 min) recovered the body lengths (line 537-542, Figure 7B, Supporting Figure S16). On the other hand, significant body shrinkage occurred after 15 min exposure in buffer solutions with lower osmolarity, ~126 mOsm containing 111 mM 2-butanone or ~138 mOsm containing 123 mM tetrahydrofuran and did not recover by subsequent 105 min exposure (line 543-549, Figure 7D). Based on these findings, I concluded that 2-butanone and tetrahydrofuran cause body shrinkage by mechanisms partly different from that caused by dehydration due to hyperosmolarity.

**Minor issues:**

*1. The final supplementary cartoon is speculative. They have no evidence that sensory neurons are affected at low concentrations (the worms chemotax fine). We do not know if muscles are less affected than motorneurons. I recommend removing this figure.*

I removed this figure (Supporting Figure 15 in the 1st draft).

*2. Use first person to help clarify what you did versus others. This is especially important when you mention other people’s work and then your own - both using third person in subsequent sentences. E.g. lines 116, 290*

I used first person in the part it should be state clearly what I did in this paper.

*3. Line 53. The chemicals might have effects not just by penetrating cells, but also on cells or on excreted components.*

I added the description as follows (line 52-54 in Introduction)
“External chemicals interact with proteins and lipids on plasma membranes, as well as extracellular biological molecules, such as the extracellular matrix, and can cause toxic effects.”

*4. Given that worms respond to ethanol very differently with different buffers (see PMID: 22486589), please make sure to mention which buffer was used. Also clarify if worm was exposed to chemical in liquid buffer or with chemical in the agar substrate with air above, which also causes different drug effects. Note supply source for agar.*

I used the same buffer solution to dissolve chemicals for exposing worms to the chemicals in all experiments. The composition of the buffer solution has been described in Materials and Methods (line 157-158). I named this buffer KOP_4_ buffer. In the first paragraph of the Results section, I clearly stated that “To assess the effects of exposure to organic solvents, the solvents were dissolved in KPO_4_ buffer solution, and *C. elegans* were soaked in buffer for 15, 30, 60, or 120 min (Materials and Methods).” (line 257-259)

*5. Please make it more apparent how the immobile state is defined. How is this different than the pause state?*

The present study does not discriminate between pause and immobile states. To avoid confusion, an absolute value below 30 µm/s was defined as an immobile state (line 180 in Materials and methods, line 291-292 in Figure 1 legend) and determined the proportion of the immobile state to observe the extent of immobility after chemical exposure. The phrase “pause” is deleted. To assess the toxic effect of alcohol on mobility, the minimal concentrations that the proportion of the immobile states reached to >50% have been compared between exposed alcohols (Figure 1D, Supporting Figure 1A, Figure 2A).

*6. Figure 1: please add units to y-axis, adjust fonts for all graphs in all panels of all figures to be more uniform and readable. For B, the situation is hard to understand without additional information. "Soaking in a buffer" on the x axis suggests that worms were soaked thrashing in buffer BEFORE measuring their crawling speed on dry agar plates. Is this the case? Explain when worms were soaked in buffer, for how long, and when did recording behavior happen. What was the buffer? Were there more than one buffer? Why not make the pause area to be a gray box rather than “colorless”? In legend, why is buffers plural here? Was there more than one type of buffer? If so, explain. Lastly, "exposure without bz" is confusing as written. It is more accurate and simpler to write "average value for untreated worms"*

- I added the units to y-axis in all figures. I have used the Arial font for all graphs in all panels of all figures.

- For panel B, I added a gray box for the “immobile” area.

- For panel C, I added a description below in the figure legend.
“Violin plots of locomotion speeds on an agar plate after soaking in KPO_4_ buffer. The x-axis represents the soaking time before locomotion assay on an agar plate.”
I used one kind of buffer solution, KPO_4_ buffer, for soaking procedure. Therefore, I replaced “buffers” to “KPO_4_ buffer" in this description.

- For panel D, I replaced “exposure without bz” to “average value of the untreated control”.

*7. Figure 2: rate? per unit time? Isn't this a proportion? Figure 2 is confusingly laid out. Please label all panels A-D. Confusing on what end point(s) is for B and C. The author has an opportunity to test for significant change in R^2 value for these models in B and C. Might be able to say that lipid solubility is significantly better model.*

- Immobility is expressed as the proportion of the immobile states. I corrected “immobility rate” to “Immobility” and stated “The proportion of the immobile state (A)” in the figure legend of Figure 2.

- I labeled all panels A-D according to the suggestion.

*8. Line 331: recovering in liquid? Or on agar plate? Please clarify. What buffer?*

I clearly stated that “the locomotion of nematodes was assessed after recovery in buffer solution without organic solvent. Nematodes were exposed to organic solvents for 2 h at concentrations that caused complete paralysis and were then soaked in KPO_4_ buffer solution without the solvents. (line 390-393)

*9. Line 357: Please cite the original data in PMID: 14675531 and PMID: 15182714*

I cited the original paper instead of the review paper. (line 415).

*10. Line 365: please put into context with how worm can respond to these chemicals with the use of smell. See 1993 Cell paper PMID: 8348618*

As the response to the main issue-1 of the reviewer #1, I added a new clause about what extent chemosensory functions are related to locomotion changes after organic solvent exposure (line 448-486). In the first sentence in this clause, I added the introduction about how worms can respond to volatile organic solvents, such as alcohol, ketone, and ether (line 450-452). I also added a description of how *C. elegans* responds to 2-butanone to promote attraction to 2-butanone in the next paragraph (line 477-480) and the detail is in the discussion section (line 673-677). And then I explained the difference between prolonged changes in amplitude of body bending after 2-butanone exposure and acute behavioral changes as observed in chemotaxis responses (line 480-486 in Results, line 677-681 in Discussion).

*11. Line 376: it is hard to conclude this here. The effect could also be on muscle tone, on hydration, and/or even on their behavioral excitement or repulsion responses that one would expect to change their posture.*

I deleted this sentence to avoid confusion.

*12. Line 414; clarify here that you know that the solvent treatment did not impact sensation or locomotion because your control group could still move to salt.*

I stated that “Therefore, the NaCl sensation was not largely affected by exposure to organic solvents at doses that did not cause any significant defects in locomotion speed.” (line 580-582).

*13. I do not understand why the author tested eat-4 as a control. They cannot perform plasticity. What was to be learned?*

I deleted the experiment using *eat-4* mutants to avoid confusion.

*14. Line 490: keep in mind that even if the chemicals cannot penetrate the worm, it can smell these chemicals and change its locomotory behavior in response. This would be reflected in changes in body posture.*

I deleted the discussion about the possible effect of motor neurons after organic solvent exposure because it is not beyond speculation. Instead, I added a discussion about the sensory neuron-mediated effect on body bending after organic solvent exposure in line 669-685.

*15. Line 528-530: this doesn't appear to be the general case because the solvents did not affect normal chemotaxis. The solvents somehow interrupted the plasticity mechanisms. Actually it is remarkable that they could chemotaxis fine after exposure to these chemicals, because like you write, the ASE neurons should be affected – presumably negatively. But they chemotaxed fine. Perhaps it is because worm has adapted to live in compost with rotting material and organic solvents?*

I agree with the notion that the salt sensing function is less susceptible to organic solvents whereas organic solvents somehow interrupted the plasticity mechanism. I added a discussion as follows.

“In the present study, I demonstrated that low-dose organic solvents cause defects in salt chemotaxis learning, whereas they did not cause gross abnormalities in NaCl sensation. Therefore, organic solvents may have suppressive effects on the mechanisms of neuronal plasticity after NaCl sensation.” (line 732-736)

*16. Line 542. a number of relevant studies on toxicity are not cited and put in context. Use pubmed to search for "elegans toxic organic solvent" or more*

I added the studies listed below.

- Related to the effects of organic solvents on sensory cilia, the toxic effect of 3-octaone on sensory cilia (line 725-732). (PMID: 36652525, 32123065)
- Effects of anesthetics on several kinds of behavior, such as male mating, egg laying and mechanosensation as well as chemotaxis, in *C. elegans* (line 739-740). (PMID: 8873562)
- Toxcity studies for toluene, a volatile organic compound known as an environmental pollutant (line 757-759). (PMID: 5033616)

**Response to Reviewer #2:**

*1. Title: Suggest change it to: “High-throughput assessment of general and specific adverse effects of organic solvents on behaviors in Caenorhabditis elegans”.*

I appreciate the important suggestion. I changed the title as follows according to the suggestions from reviewers #1 and #2.

“High-throughput assessment of the behavioral responses to toxic organic solvents in *Caenorhabditis elegans*”

*2. Abstract: Please specify the chemical showing toxic effects on behavior. In addition, the examined doses of chemicals should be indicated.*

As the reviewer pointed out, it is important to obtain the information necessary for many readers from the abstract. It was difficult to list each chemical and each dose that induced toxic effects in the abstract, but I specified the chemical groups, such as alcohols, ethers and ketones, and each of ranges of concentrations that affect speed and posture during locomotion in the abstract.

*3. Discussion: In this study, besides locomotion, the learning was also assessed. Please discuss the possible value and limitation of developed method while assessing other types of complex behaviors.*

I added the description as follows in Discussion according to the reviewer’s suggestion (line 739-754).

“It has been reported that anesthetic exposure affects several behaviors, such as male mating, egg laying, mechanosensation, and chemotaxis, in *C. elegans*. In general, distinct mechanisms are used to exert different types of behaviors. Thus, observation of multiple behaviors provides an opportunity to understand multiple aspects of toxic effects on the nervous system. In addition to innate behaviors, several paradigms of learned behavior have been developed in *C. elegans*. For example, *C. elegans* memorize external environmental cues such as chemicals and temperature during feeding and show learned attractive responses to these cues, which last for several hours. Nematodes also form long-term memory that lasts for >24 h through repeated training of associations between chemical cues and feeding experience. In future studies, it will be interesting to investigate the effects of toxicants on these complex behaviors and the underlying molecular and cellular mechanisms. These studies can then be applied to the toxicity assessment of higher organisms with complex nervous systems. As a limitation of research using *C. elegans*, the lifespan of wild-type *C. elegans* is 2–3 weeks under standard laboratory conditions, making it difficult to study the effects of chemical exposure over months or years in one generation.”

*4. Discussion: During the past 10 years, besides for molecular toxicology, one of important value of C. elegans is used to assess pollutants at environmentally relevant concentrations (ERCs). Please discuss the possible value of developed method at this aspect.*

I added the description as follows in Discussion according to the reviewer’s suggestion (line755-766).

“The high-throughput toxicity assay developed in this study can also be used to test the toxicity of environmental pollutants, such as volatile organic compounds (VOCs), heavy metals, and nanoparticles. Toluene, a VOC, has adverse effects on locomotion differently than alcohol, and the underlying molecular mechanism is postulated to be different from that of alcohol in *C. elegans*. The toxic effects of heavy metals and metal nanoparticles on the nervous and reproductive systems have been studied in *C. elegans*. The effects of environmental chemicals, such as bisphenol A and nanoplastics, which are widely present in the environment, on nematodes are also being studied. Hydrocarbons, such as toluene, are insoluble in water, making it difficult to expose nematodes to these chemicals in a buffer solution as reported in this study. By using methods for exposing toxicants from air, high-throughput assessments of chemical toxicities can be performed more broadly in the future.”
